# MARITAL TRAJECTORIES, RACE/ETHNICITY, NATIVITY, AND CARDIOVASCULAR COMORBIDITIES AMONG US ADULTS

**DOI:** 10.1093/geroni/igae098.4023

**Published:** 2024-12-31

**Authors:** Maki Karakida, Jeffrey Stokes, Qian Song

**Affiliations:** University of Massachusetts Boston, UMass Boston, Massachusetts, United States; University of Massachusetts Boston, Boston, Massachusetts, United States; University of Massachusetts Boston, Boston, Massachusetts, United States

## Abstract

Marital loss – one of the most stressful and emotional life events – often ties to negative cardiovascular health outcomes, which can be comorbid with other chronic conditions, like diabetes. Cardiovascular complications, spousal bereavement, and grey divorce are increasingly common in older adulthood, yet their effects may vary among US-born and foreign-born racial-ethnic minorities. The present study examines the longitudinal relationships between marital quality, race/ethnicity, nativity, and cardiovascular comorbid conditions among 6,953 Americans aged 58 and over. Leveraging the RAND HRS Longitudinal File 2020 (2010–2020) and binomial logistic regressions, we analyzed how married or partnered adults in 2010 undergone marital loss and developed heart disease, high blood pressure and hypertension, stroke, and/or diabetes by 2020, during the COVID-19 crisis. Across all the models, the main effects highlighted that the widowed group had a significantly higher likelihood of cardiovascular comorbidities (all p < 0.026) unlike the married and divorced groups. The model, examining the intersection of race/ethnicity and nativity – interacted with marital status – revealed that compared with US-born White married adults, US-born Hispanic divorcees (OR = 4.39; p < 0.022) and foreign-born non-Hispanic Other divorcees (OR = 10.49; p < 0.038) indicated 4-10 times greater cardiovascular comorbidity risks. Findings suggest that among older nonnative and/or racial-ethnic minorities, spousal bereavement and martial dissolution/separation developed worse cardiovascular comorbid conditions, especially during the first year of the pandemic outbreaks. Cardiovascular comorbidities, interacted with race/ethnicity, nativity, and marital status, are warranted to be further investigated.

